# Hypoxia-Ischemia and Therapeutic Hypothermia in the Neonatal Mouse Brain – A Longitudinal Study

**DOI:** 10.1371/journal.pone.0118889

**Published:** 2015-03-16

**Authors:** Jennifer C. Burnsed, Raul Chavez-Valdez, Mir Shanaz Hossain, Kalpashri Kesavan, Lee J. Martin, Jiangyang Zhang, Frances J. Northington

**Affiliations:** 1 Division of Neonatology, Department of Pediatrics, Johns Hopkins University School of Medicine, Baltimore, Maryland, United States of America; 2 Division of Neuropathology, Department of Pathology, Johns Hopkins University School of Medicine, Baltimore, Maryland, United States of America; 3 Department of Radiology and Radiological Science, Johns Hopkins University School of Medicine, Baltimore, Maryland, United States of America; Imperial College London, Chelsea &amp; Westminster Hospital, UNITED KINGDOM

## Abstract

Therapeutic hypothermia is standard of care for infants with hypoxic ischemic encephalopathy. Murine models of hypoxic-ischemic injury exist; however, a well-established mouse model of therapeutic hypothermia following hypoxic-ischemic injury is lacking. The goal of this study was to develop a full-term-equivalent murine model of therapeutic hypothermia after hypoxia-ischemia and examine magnetic resonance imaging, behavior, and histology in a region and sex specific manner. Hypoxic-ischemic injury was induced at postnatal day 10 in C57BL6 mice using a modified Vannucci model. Mice were randomized to control, hypothermia (31˚C for 4h), or normothermia (36˚C) following hypoxic-ischemic injury and stratified by sex. T2-weighted magnetic resonance imaging was obtained at postnatal day 18 and 30 and regional and total cerebral and cerebellar volumes measured. Behavioral assessments were performed on postnatal day 14, 21, and 28. On postnatal day 18, normothermic mice had smaller cerebral volumes (p < 0.001 vs. controls and p = 0.009 vs. hypothermia), while at postnatal day 30 both injured groups had smaller volumes than controls. When stratified by sex, only normothermia treated male mice had smaller cerebral volumes (p = 0.001 vs. control; p = 0.008 vs. hypothermia) at postnatal day 18, which persisted at postnatal day 30 (p = 0.001 vs. control). Female mice had similar cerebral volumes between groups at both day 18 and 30. Cerebellar volumes of hypothermia treated male mice differed from control at day 18, but not at 30. Four hours of therapeutic hypothermia in this murine hypoxic-ischemic injury model provides sustained neuroprotection in the cerebrum of male mice. Due to variable degree of injury in female mice, response to therapeutic hypothermia is difficult to discern. Deficits in female behavior tests are not fully explained by imaging measures and likely represent injury not detectable by volume measurements alone.

## Introduction

Therapeutic hypothermia (TH) is standard-of-care treatment for neonates with hypoxic ischemic (HI) encephalopathy [1, 2], a condition that affects 1.5 per 1000 newborns annually in developed nations [3]. However, TH is only partially neuroprotective, decreasing death and neurodevelopmental disability by one third [4–6]. Despite TH use in standard care of infants with HIE, there is no standardized mouse model of HI and TH with which to test new adjuvant therapies. Regional variability of HI injury has been well characterized in animal models [7–10]. However, the potential regional response to TH following HI injury remains unclear in the light of contradictory reports in human imaging studies [10–14]. There is no agreement on whether there is regional selectivity in the neuroprotection provided by TH after HI injury.

Large studies in neonates have shown a higher prevalence of cerebral palsy in males [15, 16]; however, no preclinical or clinical studies have been powered to detect a sex differences in response to TH. Rodent models have significant sex dimorphism in injury mechanisms and cell death pathways [17–23], but none of these studies have looked specifically at response to TH stratified by sex. It is unknown whether sex has an effect on response to treatment with TH in either clinical practice or translational models.

Studies combining the modified Vannucci model in mice and TH vary in age of injury, length and depth of hypothermia treatment, and outcomes investigated [23–26]. Existing studies however, have not addressed in a standardized manner, the longitudinal evolution of HI brain injury and response to TH and the influence of sex. The goal of this study was to develop a full-term-equivalent murine HI model of TH, which allows longitudinal and regional sex-specific characterization of brain injury using MRI, behavioral testing, and neuropathology. We hypothesized that TH would provide regional and sex-specific neuroprotection that will persist in this model at later stages of brain development preserving cerebral and cerebellar growth.

## Methods and Materials

Animal studies were performed with approval of the Institutional Animal Care and Use Committee at Johns Hopkins University School of Medicine and carried out with standards of care and housing in accordance with the National Institutes of Health Guide for the Care and Use of Laboratory Animals, US Department of Health and Human Services 85–23, 2011.

### Hypoxic Ischemic Injury and Hypothermia

HI injury was done on postnatal day (p) 10 C57BL6 mice (Charles River Laboratories, Wilmington, MA) as described previously using the Vannucci model adapted to mice (permanent unilateral right carotid artery ligation, plus 45 minutes of hypoxia at FiO2 = 0.08) [27, 28]. For carotid ligation surgery, mice were anesthetized using inhaled isoflurane (3% for induction, followed by 1% maintenance). Pups were returned to their dams for a 1h rest period between surgical ligation and hypoxia.

The brain of a postnatal day 10 mouse correspond in many respects, including grey matter response to injury and abundance of NMDA and AMPA receptors in the hippocampus relative to adults, to that of a term infant [29, 30]. Postnatal day 18 is pre-weaning and is considered to roughly correspond to childhood and postnatal day 30 to adolescence [30].

Immediately following HI, pups were randomized to exposure to TH (31°C) or normothermia (NT, 36°C) for 4h. Pups were placed in dry plexiglass containers, which were open to air and seated in a microprocessor controlled water bath (Thermo Fischer Scientific, Marietta, OH) to control ambient temperature in the chambers. Each subdivided chamber contained two mice. Continuous core body temperature monitoring (Ad Instruments, Inc., Colorado Springs, CO) was employed for one pup per group using a tissue implantable thermocouple microprobe placed in the rectum prior to hypoxia and continuing throughout the 4h hypothermia/normothermia period. Following the HT/NT period, pups were returned to the dam. Postnatal day 10 mice have a nesting temperature of 36°C and when in a cool room (air temperature 25°C) will rewarm at a rate of 0.1 to 0.2 degrees per minute for the first five minutes after returning to the nest [31]. Based on this, mice undergoing TH to 31°C would rewarm to 36°C in roughly 25 to 50 minutes.

Mice assigned to the control group were exposed to inhaled isoflurane on p10 for five minutes at similar concentrations as described above for the TH/NT groups and then returned to dams. Sham surgery was not performed on control mice, only anesthetic exposure. Repeat exposures to isoflurane were performed at p18 and p30 during MRI of these mice by same procedure as with HI mice.

The PI and technical staff monitored post-operative animals for activity, appetite, behavior, surgical wound and respiratory status. We did not expect to see any signs of pain or distress in relation to this procedure. However, we monitored animals daily for signs of increased or decreased respiratory rate, decrease of activity, loss of appetite, isolation from littermates, which may indicate pain or distress in the animal. No animals in this study required use of analgesia post-operatively for pain. Pups that were injured or neglected by the dam were withdrawn from the study.

### Behavioral testing

#### Negative geotaxis, open field, forelimb grasp, and air righting reflex

On p14, behavioral testing was performed including: i) negative geotaxis to evaluate labyrinthine reflex, strength, and coordination; ii) open field to evaluate locomotion and pivoting behavior, iii) air righting to evaluate for postural righting and coordination, and iv) forelimb grasp to evaluate strength. Testing was performed between the hours of 2 and 5 PM in the same quiet testing room for each group. Tests were performed as previously described [32]. Each mouse was weighed prior to testing. During the negative geotaxis task, pups were placed facing downwards on a 45° incline. Time to right upward on the incline was measured; a time over 30s was considered a failure. During open field testing, pups were placed in the center of a 13 cm circle and time to place all four paws outside circle was measured. Greater than 30s to reach outside of circle was considered a failure. During forelimb grasp test, pups were placed on wire suspended over soft surface; time of forelimb grasp was measured. For the air righting test, pups were suspended supine, 13 cm over a soft surface and dropped. Positive air righting was counted if the pup landed prone. Following behavioral testing, mice were returned to the dam.

#### Y-maze

On p21, phase 1 of Y-maze testing was performed to assess working memory. Mice were placed in one arm of the Y-maze apparatus; starting arms were alternated in a semi-random fashion for each mouse. Arm alternations were counted over a 5-minute testing period and calculated as a percentage of the total arm entries, as previously described [33].

Seven days after phase 1, phase 2 was performed to test to study spatial and recognition memory. Mice were allowed to explore Y-maze for 5 min with one arm blocked randomly. After a 20 min rest period, animals were allowed to freely explore the maze for five minutes while number of entries and time spent in each arm were recorded [34].

### Magnetic resonance imaging (MRI)/analysis

In vivo MRI of the mouse brains at p18 and 30 were performed on a horizontal 11.7 Tesla MR scanner (Bruker Biospin, Billerica, MA) equipped with triple-axis gradient (maximum gradient strength = 74 Gauss/cm) using a volume excitation coil and a 4 channel phased array mouse head receive-only coil. Animals were anesthetized with 1% to 1.5% isoflurane in a mix of oxygen and air at 1:3 ratio and placed in an animal holder (Bruker Biospin, Billerica, MA). Respiration was monitored using a pressure sensor (SAII, Stony Brook, NY) and maintained at 50–60 breaths per minute by adjusting the concentration of isoflurane. Multi-slice T2-weighted images were performed utilizing a rapid acquisition with refocused echoes (RARE) sequence with the following parameters: echo time (TE)/repetition time (TR) = 60/3800 ms, RARE-factor = 8, four signal averages, field of view (FOV) = 16 mm x 16 mm, 36 slices with 0.4 mm slice thickness, in-plane resolution of 0.06 mm x 0.06 mm, and an imaging time of 12 minutes. All animals recovered within 5 minutes after imaging.

Volumetric measurements were obtained from T2-weighted images using Amira software (http://www.vsg3d.com). The researcher performing measurements was blinded to sex and treatment. Total residual cerebral volume (total brain volume—cerebellar volume—lesion volume), total cerebellar volume, and contralateral and ipsilateral regional volumes (cortex, hippocampus, striatum, and thalamus) were measured in mm^3^. These regional volumes were used to calculate percentage injury [(contralateral volume—ipsilateral volume)/contralateral volume]*100. Residual cerebral and cerebellar growth from p18 to p30 were calculated as a percentage of volume increase relative to p18 volume of the region for a subset of animals that had serial MR images at p18 and p30.

### Histology

On p18 or p30, mice were deeply anesthetized with inhaled isoflurane via the one-drop exposure method, during which a small piece of gauze with a drop of isoflurane on it was placed in the tip of a 3ml syringe. This was placed over the animal’s nose and provided inhaled anesthesia during cut-down in preparation for intracardiac perfusion. The mice were then euthanized by exsanguination with cold 0.1M phosphate buffered solution (PBS, pH 7.4, Amresco, Solon, OH) via intracardiac perfusion, and then perfused with 4% paraformaldehyde in 0.1M phosphate buffered saline for 15 minutes at 4 ml/minute for brain fixation. Brains were removed and postfixed in PBS for storage, then placed in paraformaldehyde for 48 hours prior to processing. Specimens were processed at the Tissue Microarray Laboratory at Johns Hopkins Medical Institute in Baltimore, MD. The brains were processed in an automated tissue processor (Tissue-Tek VIP 2000, Sakura Finetek, Torrance, CA) for paraffin embedding of tissue. The paraffin embedded brains were then sectioned into coronal slices of 5μm thickness and mounted on glass microscope slides. Selected slides were stained with hematoxylin and eosin (H&E) using a Leica AutoStainer XL (Leica Biosystems, Richmond, VA). Staining with Fluorojade C (Chemicon, Temecula, CA) with DAPI co-staining was performed using previously described methods [35]. Slide immunostaining on the paraffin embedded tissue sections was done following deparaffinization with xylene, rehydration with ethanol, and heat antigen retrieval with at 60°C for 60 minutes and antigen retrieval with Proteinase K. Primary antibody was incubated on sections overnight. Glial fibrillary acidic protein (GFAP) antibody (Dako, Denmark) was used at 0.06 μg/μl and anti-neurofilament M (Millipore, Temecula, CA) was used at 0.05 μg/μl. Alexa Fluor 488 goat anti-rabbit antibody (Molecular Probes, Grand Island, NY) was used as secondary for GFAP. Goat anti-rabbit antibody (MP Biomedical, Aurora, OH) was used as secondary for neurofilament M and developed following a standard diaminobenzidine (DAB) reaction.

### Statistical analysis

Statistical analysis was performed using one-way ANOVA with Tukey post-hoc analysis for MRI volumes and growth rates from p18 to p30, Y-maze test, open field test, negative geotaxis, and forelimb grasp test. Results were represented as box and whisker plot, where the 25th and 75th percentiles limited the box and the solid line represented the median. Air righting behavior was analyzed using likelihood ratio test. Analysis was performed by sex-stratification and significance was assigned to a p < 0.05. IBM SPSS Statistics 18.0 software (IBM Corporation, Armonk, NY) was used for analysis.

## Results

### Animals

A sample size of 9 to 15 C57BL6 mice per sex, treatment, and time point was used and a total of 180 mice were included in the study. The mortality rate for this model was 15% (55.6% females, 44.4% males, X^2^ = 0.33, p = 0.59). Seventy percent of deaths occurred during the hypoxia phase of the experiment. One surviving NT male was excluded from analysis because of a total brain infarct resulting in cystic degeneration of the entire cerebrum. Body weight was not significantly different between sexes when measured at p14, though the TH males had significantly lower weight compared to male controls (p = 0.009). Mice being treated with TH reached goal temperature of 31°C ± 0.77° within 15 minutes after starting cooling and remained there for the duration of the 4 hour period. NT mice were at 35.6°C ± 0.50° and remained there throughout the 4 hour period. Temperatures of the two groups differed within 15 minutes of the start of cooling (p<0.001).

### Behavioral testing

**Fig 1:**
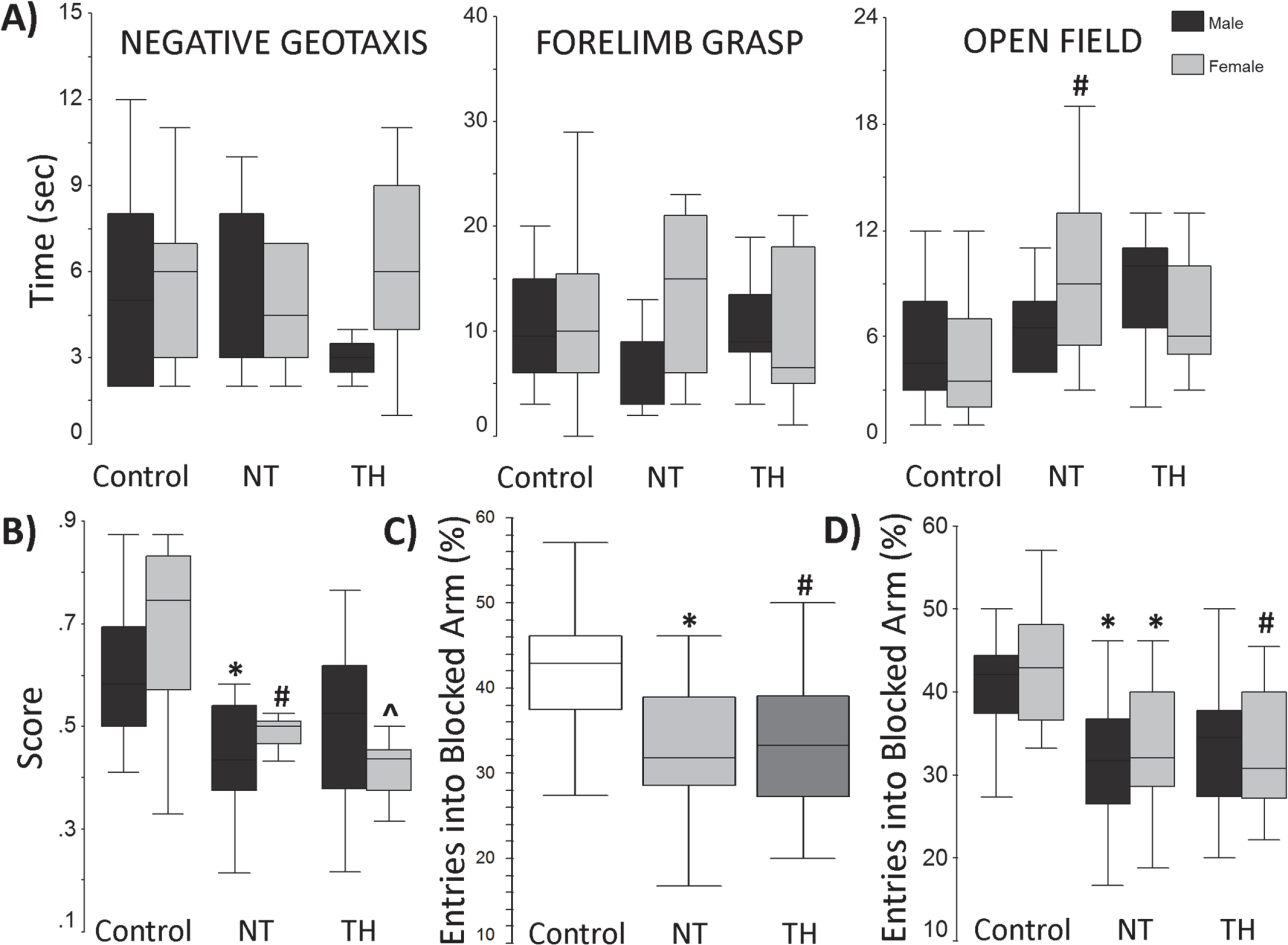
Behavioral testing. **A)** Negative geotaxis task and forelimb task times at p14 were similar amongst treatment groups in both sexes. NT female mice performed open field test slower than control females (#p = 0.04). Males: control n = 14, NT n = 10, TH n = 11, females: control n = 15, NT n = 10, TH n = 10. **B)** Y-Maze testing at p21 (Phase 1): NT males scored lower than controls (*p = 0.02) and both NT and TH females scored lower than controls (# p = 0.01 and ^ p = 0.002, respectively). **C)** Y-maze Phase 2 (p28). Both NT and TH mice entered blocked arm fewer times than controls (*p<0.001 and #p = 0.001, respectively). **D)** Y-Maze Phase 2, stratified by sex. NT and TH females entered into blocked arm fewer times than control females (*p = 0.02 and # p = 0.01, respectively). NT males entered blocked arm blocked arm fewer times than controls (* p = 0.02). Males: control n = 12, NT n = 11, TH n = 12, Females: control n = 11, NT n = 10, TH n = 11.

At p14, no differences were detected in the negative geotaxis or forelimb grasp tasks in either sex. NT female mice were slower to complete open field testing (p = 0.009 vs. female controls; Fig. 1A). NT treated male mice were more likely to fail the air righting test (p = 0.03 vs. TH treated male mice).

At p21, both NT and TH treated female mice scored lower on phase 1 of the Y-maze test (p = 0.01 and p = 0.002 vs. female control, respectively). NT treated male mice scored lower on phase 1 (p = 0.02 vs. male control mice; Fig. 1B). At p28, on phase 2 Y maze testing, both the NT and TH groups entered the blocked arm less than controls (Fig. 1C, p < 0.001 and p = 0.001, respectively). When data was stratified by sex, NT and TH females entered the previously blocked arm less frequently than control females (p = 0.02 and p = 0.02, respectively). NT males entered the previously blocked arm less than controls (p = 0.02) (Fig. 1D).

### Imaging

**Fig 2:**
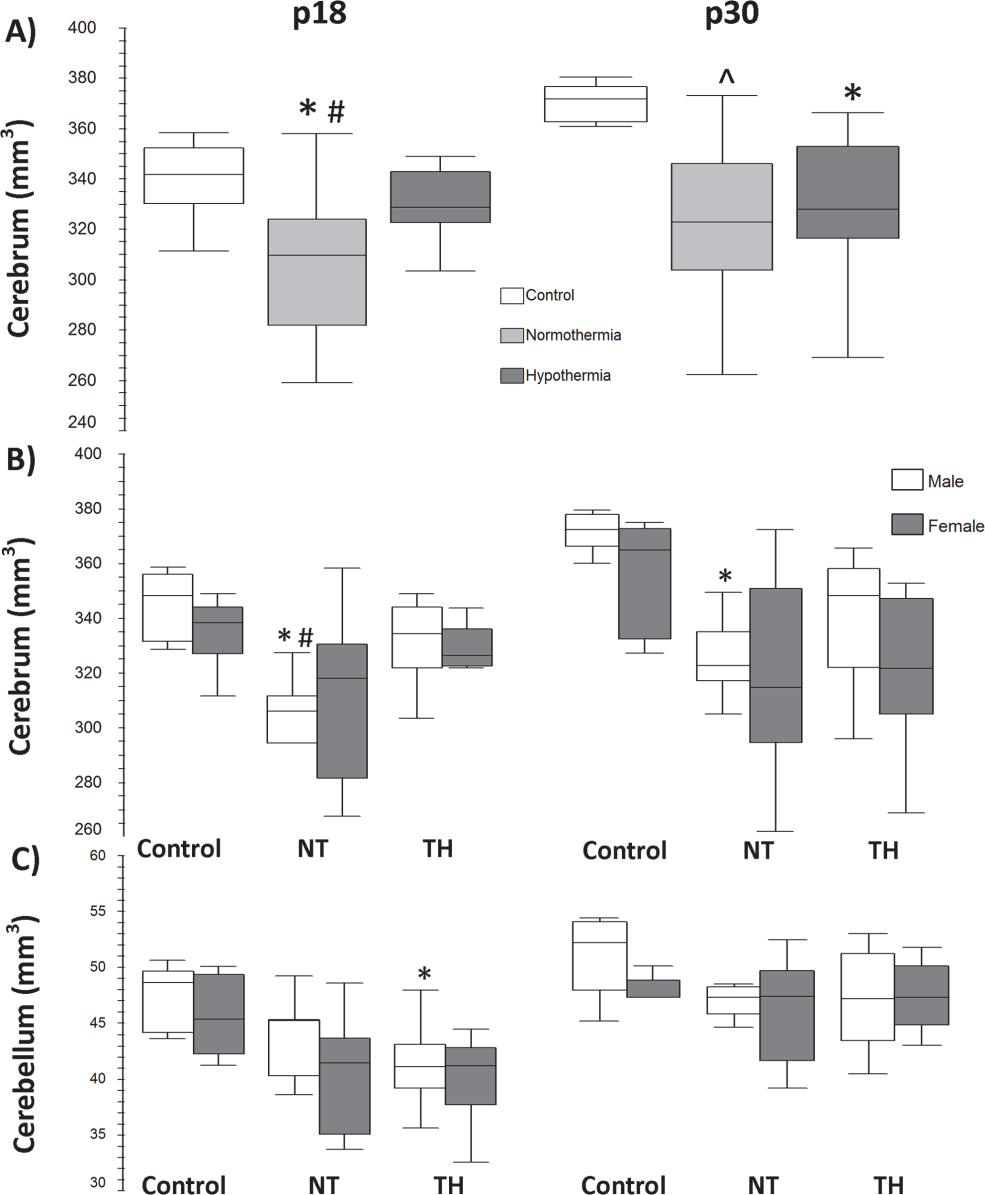
Cerebral and cerebellar volumes on MRI at p18 and p30. **A)** Residual cerebral volumes at p18 are lower in NT compared to controls (* p<0.001) and to TH (# p<0.001). By p30, both NT and TH cerebral volumes are less than controls (* p = 0.003 and ^ p<0.001). **B)** Residual cerebral volumes stratified by sex. Male mouse residual cerebral volume at p18 was smaller in NT vs. TH and controls (*p<0.001 and # p = 0.002). At p30, differences persisted in males between NT and controls (*p<0.001). No differences were found between female groups. **C)** Cerebellar volumes stratified by sex. At p18, cerebellar volume in TH males was smaller than controls (*p = 0.01). No differences between groups were found in female cerebellar volume. p18 males: control n = 6, NT n = 9, TH n = 12; females: control n = 6, NT n = 11, TH n = 10. p30 males: control n = 7, NT n = 9, TH n = 12; females: control n = 5, NT n = 11, TH n = 11.

At p18, NT treated mice had smaller residual cerebral volumes (mm^3^) than controls and TH treated mice (p < 0.001 for both comparisons) (Fig. 2A). However, at p30 both NT and TH treated groups had smaller residual cerebral volumes than did control mice (p < 0.001 and p = 0.003, respectively) (Fig. 2A). When stratified by sex at p18 and at p30, residual cerebral volume in female mice showed no significant difference between treatment groups at p18 and 30. At p18, residual cerebral volumes of NT treated male mice were reduced compared to control and TH males (p = 0.001 vs. control and p = 0.002 vs. TH) (Fig. 2B). At p30, NT male mice continued to have smaller residual cerebral volumes than male controls (p < 0.001) (Fig. 2B), while TH male mice did not differ from controls at either p18 or p30. Female cerebellar volumes were similar between groups at both time points (Fig. 2C). In males, cerebellar volumes (mm^3^) at p18 were smaller in TH mice (p = 0.01 vs. male control); this difference was not present at p30.

**Fig 3:**
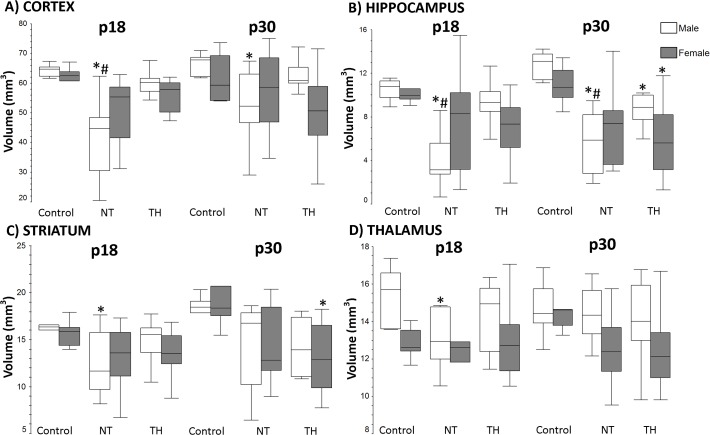
Regional volumes (cortex, hippocampus, striatum, and thalamus) on MRI ipsilateral to carotid ligation at p18 and p30. **A&B)** The cortex and hippocampus are smaller in NT compared to TH males (# p<0.001). Regional volumes were not different between female groups. NT males continue to have smaller volumes in the cortex compared to controls (* p = 0.042). At p30, the hippocampal volumes in NT males are smaller than both control and TH males but the TH group also has smaller hippocampus volume compared to control (*p <0.001 (NT), p = 0.002 (TH), # p = 0.041). **C)** NT males had smaller striatal volumes than controls at p18 (*p = 0.019). The striatum volume at p30 was smaller in TH females when compared to controls (*p = 0.034). **D)** The thalamus at p18 is smaller in male NT mice (vs. control p = 0.043), there were no differences between groups at p30.

Overall, NT treated males had smaller hippocampal and cortical volumes at both time points and smaller striatal and thalamic volumes only at p18. Females showed no regional differences at p18. Hippocampal and cortical volumes in NT treated males were smaller at p18 (p < 0.001 vs. control and p < 0.001 vs. TH; Fig. 3). Male NT mice continued to have smaller cortical and hippocampal volumes at p30 when compared to controls (Fig. 3A and B). Male NT mice had smaller striatal and thalamic volumes at p18 when compared to controls; but by p30, there were no differences between groups (Fig. 3C and D). Female mice did not have any difference between groups at p18 in any region. At p30, female TH mice had smaller hippocampal (p = 0.03) and striatal (p = 0.02) volumes than did control females (Fig. 3B and C); but these volumes were no different from NT females.

When injury was analyzed as median percent injury as described previously [36], TH was protective in males at p18 in all supratentorial brain regions analyzed (hippocampus, cortex, striatum, and thalamus) (Table 1). At p30, hypothermic neuroprotection persisted in the hippocampus, cortex and thalamus in males, with a greater percentage injury in NT compared to control and TH in each of these regions. In females, only the hippocampus was protected by TH at p18 in this analysis. However, this was transient with NT versus TH differences dissipating by p30 in females (Table 1). At p30, TH and NT females had greater injury in the hippocampus and striatum than controls (Table 1), while only TH females had greater injury in thalamus than controls (Table 1).

**Table 1:** Percentage injury at p18 and p30.

Sex	Region	Treatment Group	Median % Injury at p18 (Interquartile Range)	Significance (p)	Median % Injury at p30 (Interquartile Range)	Significance (p)
**Male**	Hippocampus	Control	7.10 (0.67–11.44)	p < 0.001*	1.09 (-0.89–6.17)	p < 0.001*
		Normothermia	73.33 (54.02–85.5)	—	67.07 (44.69–84.67)	—
		Hypothermia	14.6 (9.53–28.91)	p < 0.001*	29.99 (18.57–51.17)	p = 0.01*, p = 0.002†
	Cortex	Control	7.93 (3.68–11.01)	p = 0.001*	4.07 (2.38–6.49)	p = 0.008 *
		Normothermia	37.04 (24.67–53.59)	—	28.29 (15.34–43.72)	—
		Hypothermia	8.8 (4.09–14.9)	p = 0.001*	8.85 (2.21–23.6)	p = 0.05*
	Striatum	Control	3.56 (0.43–8.16)	p = 0.003*	2.02 (-1.19–7.35)	p = 0.04*
		Normothermia	34.84 (18.93–43.79)	—	20.62 (3.4–50.12)	—
		Hypothermia	14.46 (7.3–27.89)	p = 0.04*	18.11 (9.79–30.66)	—
	Thalamus	Control	0.00 (-0.21–0.47)	p = 0.002*	0 (-0.14–0.61)	p < 0.001*
		Normothermia	7.13 (4.83–12.33)	—	15.65 (5.19–20.68)	—
		Hypothermia	3.01 (-0.4–8.31)	p = 0.04*	7.25 (5.09–8.6)	p = 0.04*, p = 0.02†
**Female**	Hippocampus	Control	3.23 (0.3–5.05)	p = 0.04*	0 (-10.56–26.15)	p = 0.03*
		Normothermia	71.91 (15.8–96.92)	—	51.4 (33.72–70.55)	—
		Hypothermia	55.72 (23.35–81.08)	p = 0.06*	70.12 (48.9–81.82)	p = 0.005†
	Cortex	Control	8.31 (1.81–13.3)	p = 0.19‡	4.86 (0.76–6.8)	p = 0.07‡
		Normothermia	36.72 (9.8–53.19)	—	20.03 (10.4–35.49)	—
		Hypothermia	25.41 (3.06–47.6)	—	31.1 (18.53–50.65)	—
	Striatum	Control	0.26 (-3.05–7.66)	p = 0.04*	0 (-1.18–2.51)	p = 0.02*
		Normothermia	41.76 (20.59–56.19)	—	23.57 (14.29–40.18)	—
		Hypothermia	25.16 (7.29–43.57)	—	33.22 (16.67–51.02)	p = 0.01†
	Thalamus	Control	2.15 (-0.66–7.02)	p = 0.09‡	0.94 (-0.7–3.9)	—
		Normothermia	10.74 (5.28–22.4)	—	7.47 (3.64–10.9)	—
		Hypothermia	7.02 (4.63–10.75)	—	7.05 (4.02–16.26)	p = 0.02†
**Sex**	**Region**	**Treatment Group**	**Median % Injury at p18 (Interquartile Range)**	**Significance (p)**	**Median % Injury at p30 (Interquartile Range)**	**Significance (p)**
**Male**	Hippocampus	Control	7.10 (0.67–11.44)	p < 0.001*	1.09 (-0.89–6.17)	p < 0.001*
		Normothermia	73.33 (54.02–85.5)	—	67.07 (44.69–84.67)	—
		Hypothermia	14.6 (9.53–28.91)	p < 0.001*	29.99 (18.57–51.17)	p = 0.01*, p = 0.002†
	Cortex	Control	7.93 (3.68–11.01)	p = 0.001*	4.07 (2.38–6.49)	p = 0.008 *
		Normothermia	37.04 (24.67–53.59)	—	28.29 (15.34–43.72)	—
		Hypothermia	8.8 (4.09–14.9)	p = 0.001*	8.85 (2.21–23.6)	p = 0.05*
	Striatum	Control	3.56 (0.43–8.16)	p = 0.003*	2.02 (-1.19–7.35)	p = 0.04*
		Normothermia	34.84 (18.93–43.79)	—	20.62 (3.4–50.12)	—
		Hypothermia	14.46 (7.3–27.89)	p = 0.04*	18.11 (9.79–30.66)	—
	Thalamus	Control	0.00 (-0.21–0.47)	p = 0.002*	0 (-0.14–0.61)	p < 0.001*
		Normothermia	7.13 (4.83–12.33)	—	15.65 (5.19–20.68)	—
		Hypothermia	3.01 (-0.4–8.31)	p = 0.04*	7.25 (5.09–8.6)	p = 0.04*, p = 0.02†
**Female**	Hippocampus	Control	3.23 (0.3–5.05)	p = 0.04*	0 (-10.56–26.15)	p = 0.03*
		Normothermia	71.91 (15.8–96.92)	—	51.4 (33.72–70.55)	—
		Hypothermia	55.72 (23.35–81.08)	p = 0.06*	70.12 (48.9–81.82)	p = 0.005†
	Cortex	Control	8.31 (1.81–13.3)	p = 0.19‡	4.86 (0.76–6.8)	p = 0.07‡
		Normothermia	36.72 (9.8–53.19)	—	20.03 (10.4–35.49)	—
		Hypothermia	25.41 (3.06–47.6)	—	31.1 (18.53–50.65)	—
	Striatum	Control	0.26 (-3.05–7.66)	p = 0.04*	0 (-1.18–2.51)	p = 0.02*
		Normothermia	41.76 (20.59–56.19)	—	23.57 (14.29–40.18)	—
		Hypothermia	25.16 (7.29–43.57)	—	33.22 (16.67–51.02)	p = 0.01†
	Thalamus	Control	2.15 (-0.66–7.02)	p = 0.09‡	0.94 (-0.7–3.9)	—
		Normothermia	10.74 (5.28–22.4)	—	7.47 (3.64–10.9)	—
		Hypothermia	7.02 (4.63–10.75)	—	7.05 (4.02–16.26)	p = 0.02†

* = vs. NT

† = vs. ctrl

‡ = between groups

**Fig 4:**
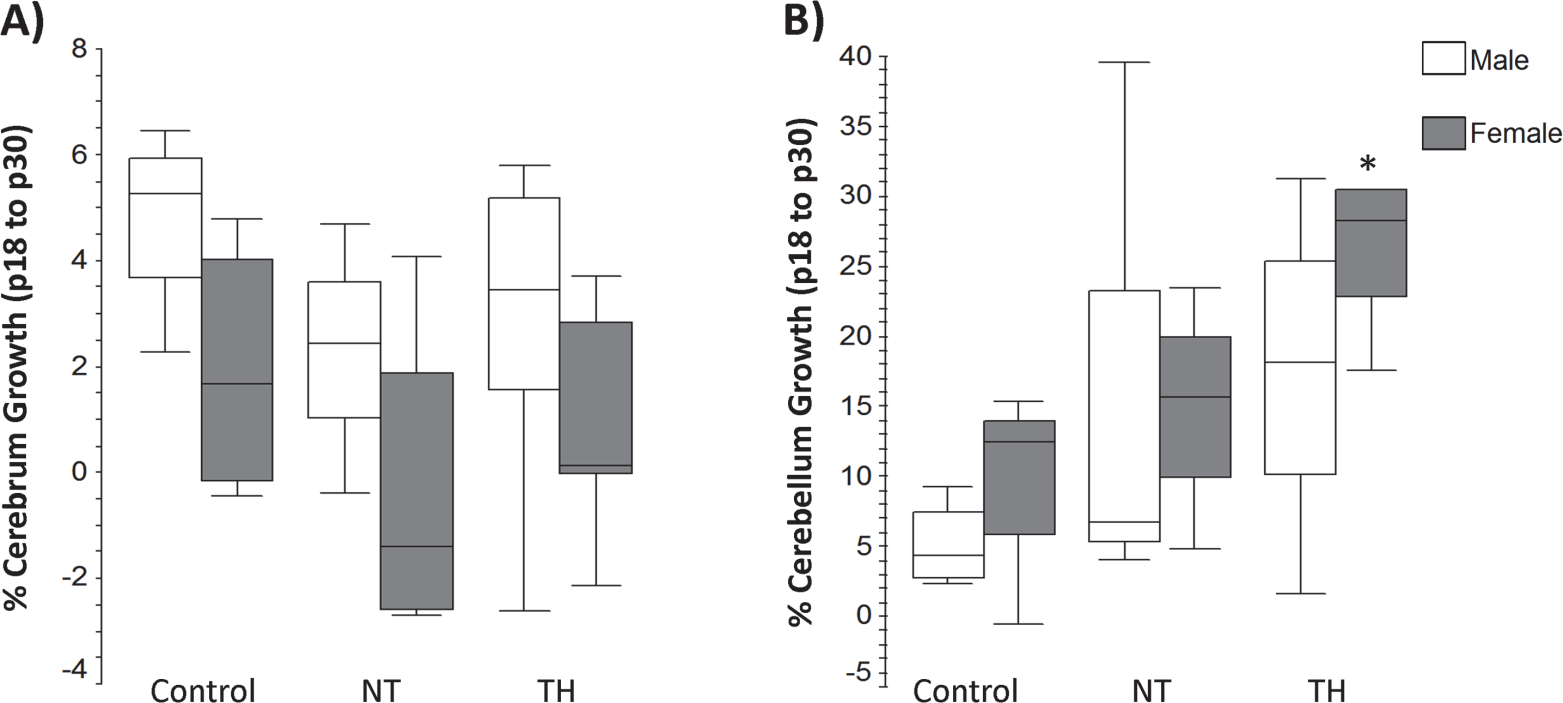
Repeated measures of cerebral and cerebellar volume analyzed by total percentage of growth from p18 to p30. **A)** There were no significant differences in cerebral growth amongst groups. **B)** Cerebellar growth in male mice was not different between treatment groups. Female TH treated mice had significantly increased cerebellar growth when compared to control female mice (*p = 0.048). Controls n = 4 males and females, NT n = 4 males and females, TH n = 6 males and 5 females.

As a measurement of growth, percentage of change in residual cerebral volume from p18 to p30 (Fig. 4A), showed no difference in either sex. Injury and treatment with TH appeared to accelerate cerebellar growth from p18 to p30 especially in females. Rate of growth in TH female mice was greater than controls (p = 0.05) (Fig. 4B). Males did not have any differences in cerebellar growth between treatment groups.

**Fig 5:**
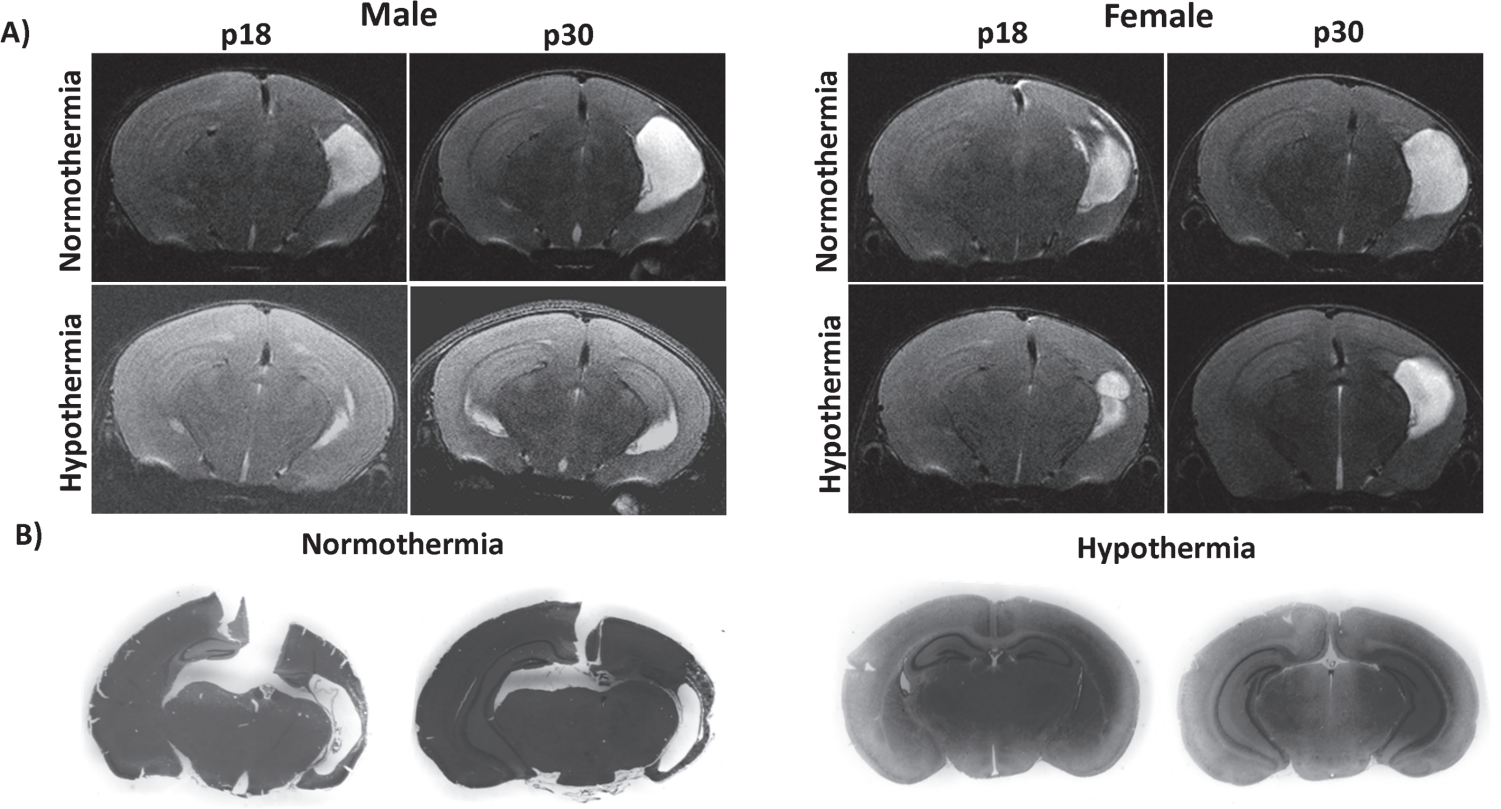
Representative MRI and gross histopathology at p18 and p30. **A)** Representative T2—weighted MR imaging animals at each time point, treatment, and sex. Images at p18 and p30 are from the same animal. The trend of mild to moderate, variable injury and neuroprotection particularly in females is demonstrated. **B)** Low power views H&E stained anterior and posterior sections from p30 male mice.

Representative MRI images from each treatment and sex group and gross histology from each treatment group are displayed in Fig. 5A-B.

### Neuropathology

**Fig 6:**
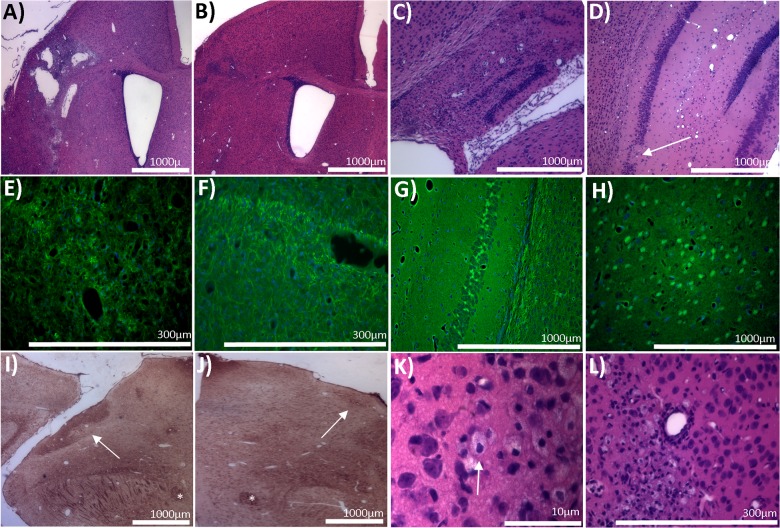
Neuropathology in HI mice at p18. **A)** Severe injury in cerebral cortex with focal cystic lesion at a mid-striatal level. **B)** Milder cortical injury (compared to A), a focal gliotic scar marking an area of neuronal loss. **C)** Severe hippocampal injury but with preservation of overall architecture of hippocampus. Pyramidal neuron elimination is prominent and remnants of the granule cell layers in dentate gyrus remain. The hippocampus is infiltrated with small cells. **D)** Mild hippocampal injury observed as small foci of pyramidal neuron loss (arrow). **E)** Fluorojade positive neuritic processes within the ipsilateral cortical neuropil. **F)** Fluorojade positive processes near the hippocampal alveus. **G)** Fluorojade positive cells and processes within the pyramidal layer of the contralateral hippocampus and the overlying corpus callosum. **H)** Fluorojade positive cells in the ipsilateral amygdala. **I&J)** Neurofilament immunostaining of the contralateral cerebral peduncle (I), containing corticospinal tract axons, and the ipsilateral cerebral peduncle (J) showing a loss of axonal neurofilament immunoreactivity. Ipsilateral cortex was severely injured. Asterisks identify columns of the fornix for orientation. **K)** Swollen astrocytes in cortex adjacent to area of injury on H&E. **L)** Signs of ongoing inflammatory response on H&E staining with vascular cuffing and activated astrocytes.

**Fig 7:**
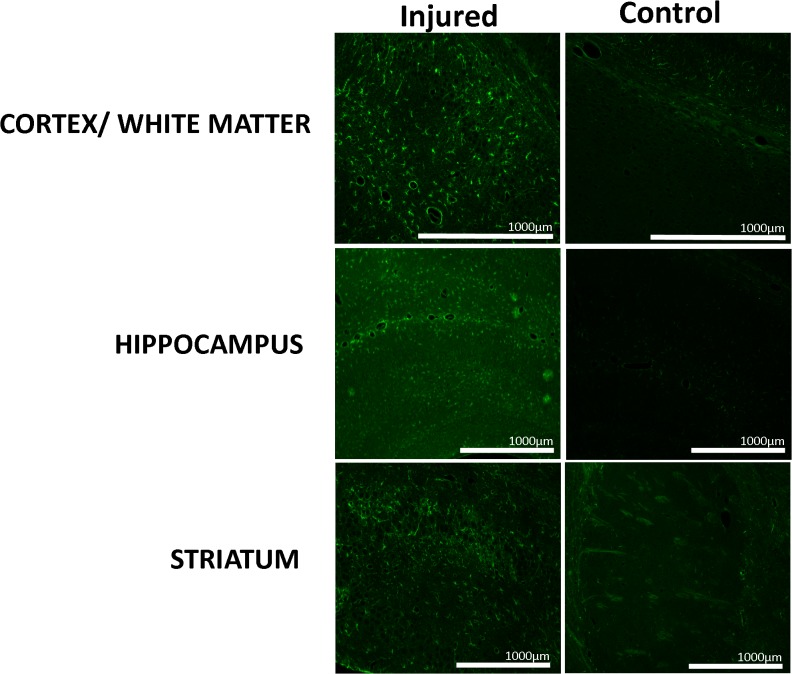
GFAP immunohistochemistry at p18. Cortex and white matter injury showing GFAP positive areas and paucity of stain in control. Hippocampal injury with glial staining and lack of staining in control mouse. Striatal injury with widespread GFAP staining indicating glial activation and lack of staining in control, other than normal white matter staining.

H&E stained sections from p30 male mice viewed macroscopically revealed severe injury in NT mice and a lesser but variable amount of injury in TH mice (Fig. 5B). Some animals, such as the p30 male in Fig. 5B, were afforded a great deal of neuroprotection with TH. Microscopic examination at p18 revealed similar neuropathologic changes in both TH and NT but degree of injury differed. NT mice had variable injury with some severe hippocampal and cortical injuries with infarct extending anteriorly to the level of the striatum on H&E staining (Fig. 6A & C) and others with minimal cortical and hippocampal injury (Fig. 6B & D). Fluorojade staining at p18 revealed many fluorojade positive processes near areas of infarct but very few positively staining cells (Fig. 6E & F). However, the contralateral cortex and hippocampus contained fluorojade positive cell staining in areas remote from sites of injury in the ipsilateral hemisphere (Fig. 6G & H). Examination of neurofilament immunostained sections revealed lack of staining ipsilateral to the lesion in the cerebral peduncle (Fig. 6J) especially in comparison to the contralateral cerebral peduncle (Fig. 6I). Signs of astrocyte activation and ongoing inflammatory response were highly evident on H&E staining and most conspicuous in severely injured brains (Fig. 6K & L). GFAP immunostained sections from p18 mice revealed ongoing glial activation and scaring ipsilateral to lesion in cortex, hippocampus, and striatum (Fig. 7).

## Discussion

Four hours of hypothermia following HI injury in this murine p10 model provided neuroprotection one week post-HI; however, neuroprotection did not persist twenty days post-HI in the group as a whole. When results were stratified for sex, neuroprotection was evident on both MRI and behavioral testing in male mice out to p30. In this model, in female mice, injury and HT neuroprotection was highly variable at all time points. When the entire female cohort was grouped, neither significant injury nor neuroprotection was evident on residual cerebral volumes measured with MRI. Percent injury differed between female treatment groups with the hippocampus and striatum injury greater in normothermia females at p18. This suggests lesser initial injury and more transient neuroprotection in females. Behavioral testing revealed deficits in working memory in injured female mice that were not recoverable with hypothermia and were consistent with a transient degree of neuroprotection.

Overall, these findings are consistent with those being reported in clinical trials of therapeutic hypothermia for the treatment of HIE. Generally, most of the large studies show overall differences in outcomes at 18–24 months of age between treated vs. non-treated neonates; however, neither the TOBY nor the CoolCap trial found a difference in the primary outcome variable, death or severe disability at 2 years, between cooled and non-cooled infants. When the groups were further subdivided and additional analyses performed, a positive effect of therapeutic hypothermia was demonstrated [37–48]. Comparisons between cooled and non-cooled infants at school age are mixed. Many studies do not reach significance [5, 49] between the groups, but others do show continued improved outcomes in school age children after therapeutic hypothermia [4]. None of the clinical studies to date have been analyzed for an effect of sex on outcomes.

MRI studies of human infants treated with hypothermia have conflicting results; some data suggest selective cortical protection [13] and others demonstrate selective basal ganglia and thalamic protection [10–12, 14]. In this study, analysis of regional volumes on MR imaging revealed that the most severe injury was to the cortex and hippocampus, consistent with the known regional vulnerability of these regions to neonatal HI [7–10]. This data indicate a widespread degree and persistence of neuroprotection for male mice with hypothermia. This may suggest generalized neuroprotection with hypothermia or selective protection to the primary sites of injury in this model of HI (hippocampus and cortex) with secondary protection of the striatum and thalamus due to preservation of connections from the primarily injured regions.

Using residual cerebral volume and regional volume as measures of injury and protection, there was no difference between control, normothermia or hypothermia females at either p18 or p30. The minor degree of protection in hypothermia treated females found using measures of lesion volume and percent regional injury measures is clearly transient, dissipating by p30. Specifically, when median residual brain volumes were examined closely, normothermia and hypothermia treated females are indistinguishable by p30. This finding is reinforced by examination of the median regional volumes at p30, as hypothermia treated females had smaller hippocampal and striatal volumes than controls and no difference in hippocampal and striatal volumes between hypothermia and normothermia treated females.

When results were expressed as percent injury, hypothermia-mediated neuroprotection was even more striking in males with significant hypothermia-mediated neuroprotection in all regions analyzed at p18; this hypothermia-mediated neuroprotection persists widely at p30 and confirms generalized rather than region-specific hypothermic neuroprotection in the mouse brain. Female results expressed as percent injury were also striking in the lack of demonstrable neuroprotection at p30 in multiple regions in hypothermia treated females. While these data are generally congruent with other rodent, piglet, and human neonatal data on the effects of therapeutic hypothermia, the sex-stratified results provide additional insight into the incomplete and variable degree of neuroprotection consistently seen in all studies of neonatal hypothermia.

Cerebellar volumes measured post-HI suggest transient injury in both sexes that fully recovers between p18 and p30, most prominently in females. Qualitative examination of cerebellar histology did not reveal a cause for this transient delay in cerebellar growth in response to HI and hypothermia, as hypothermia-exposed brains showed no obvious pathology in cerebellum. There were also no obvious ipsilateral—contralateral differences found in the cerebellar hemispheres. The cerebellum is known to be vulnerable to HI [7]; however, its response to hypothermia is not well described. Most of the literature on the effect of acquired brain injury on cerebellar development has focused on preterm infants and very young (p2) rats. Both human preterm infants and the p2 rat pup are susceptible to HI mediated disruption of all cell layers and WM of the cerebellum [50–52]. Although the p10 mouse cerebellum seems to be more resistant to injury than the p2 rat pup, the cerebellum continues to develop from p10 to p18 [53] with inward migration of the external granule cell layer among other changes. The effect of both injury and hypothermia on this continuing development is unknown and warrants further exploration. Overall, these cerebellar data suggest that there may be a transient injury or hypothermia-mediated slowing of growth before p18 that recovers most robustly in hypothermia treated females to allow normal cerebellar volumes by p30. The question of whether this transient delay and then catch up growth results in abnormalities of cerebellar function is an area that also warrants further investigation. Normothermia treated males fared worse when initially tested in the Y-maze and this result is congruent with the injury to the cortex and hippocampus. Hypothermia provided neuroprotection of memory behaviors in males in this model. The same is not true for females. Both normothermia and hypothermia females scored lower than controls on tests of working and spatial recognition memory in the Y-maze, raising the likelihood that the lower hippocampal volumes on MRI in the hypothermia treated females at p30 was significant. This is supported by the higher percentage of injury in normothermia and hypothermia treated female mice compared to controls, which corresponds to the Y-maze findings in females. This raises concern that hypothermia may not offer long-term protection in females in this model. This result is consistent with Wagner, et al. who examined p7 rats serially following HI and 26h of therapeutic hypothermia correlating behavior and infarct volume on MRI. That study reported a decrease in infarct volume and improved functional outcomes in hypothermia treated animals but all HI animals performed worse than controls [54]. This failure of behavioral memory tests in females likely reflects microstructural injury or biochemical abnormalities not fully demonstrable with MRI volume measurements alone and indicates that hypothermia may not be protective against these HI-induced changes in female mice.

The brain pathology in this model, seven days post HI, is congruent with the level of injury and neuroprotection seen with MRI in individual mice. In cases with injury, histology is notable for evidence of continued inflammatory response with both H&E and fluorojade C staining of processes and cells in remote areas. GFAP stained tissues are notable for areas of ongoing glial activation remote from ipsilateral injury. This is a well-known finding and a potential target for “late” therapies for HI. Evidence of remote continuing cell death and neurodegeneration observed seven days post HI and hypothermia is a potential target for late therapies.

The sex differences in response to HI and hypothermia could be a species or strain specific phenomenon; as few mouse models of therapeutic hypothermia have been published and no studies, preclinical or clinical, have controlled for sex in the final analysis of hypothermic-mediated neuroprotection. However, previously published sex-specific cell death pathways following HI in the rodent may explain the differences seen in response to HI and therapeutic hypothermia [17–19, 23, 55–56]. Sex differences in response to hypoxia have been demonstrated in both rodents prenatally and premature human infants and rodents postnatally [57–58]. Though few studies have been powered to examine the effect of sex on neuroprotection, in previous studies neonatal rats have exhibited superior hypothermic neuroprotection in females [59–61]. Bona, et al examined p7 rats following treatment with therapeutic hypothermia and though the study was not powered to examine differences between sex they found that females had lower neuropathologic scores and better functional outcomes compared to males. Studies by Fan, et al. and Thoresen, et al. examining adjuvant therapies to hypothermia in rat pups found that females fared better on behavioral testing when compared to males. It is unclear whether the sex differences found in the model used in this study are species or strain specific vs. true sexual dimorphism; this warrants further investigation. A subgroup of male and female mice responds to HI with infarction of the entire ipsilateral cerebrum resulting in large porencephalic cystic changes. This phenomenon accounts for some of the variability seen in the present study, as these animals were not excluded from analysis if they survived to be imaged.

A mouse model of HI has been developed [27]; however, there is not a well agreed upon model of HI and hypothermia in the neonatal mouse, especially in a more mature model. Zhu [23] performed hypothermia (34°C) during hypoxia in p7 mice. Carlsson [24] performed hypothermia to 33°C for 5 hours immediately following hypoxia or for 4 hours after a 2 hour post-hypoxia rest period with the dam in p9 mice and Liu cooled p7 mice to 31°C for 3.5 hours immediately following hypoxia. Our model most closely approximates that of Carlsson. As the same advantages accrue to a mouse model of hypothermia as do to mouse models of HI and other brain injuries, there exists a compelling need for a reproducible model of HI and hypothermia in both the p7 and p10 HI mouse models.

Strengths of this study include the development of a reproducible model of HI and therapeutic hypothermia in a mouse with examination of imaging, behavior and brain growth of the same subset of mice over several time points. The use of imaging is especially important in that MRI currently provides the best means to estimate injury and response to therapy in both translational and clinical studies. MRI also has prognostic value when predicting neurodevelopmental outcomes in infants with HIE [14, 62–64].

Importantly, the results of this study of hypothermia in a routinely used mouse model of neonatal HI mimic findings being reported in human studies of therapeutic hypothermia including, incomplete but demonstrable and persistent protection from injury. A limitation of this model is the variability of injury as measured by MRI, especially in female mice. These results include all mice that survived to imaging in the analysis. Had animals with an apparent hemispheric infarct been excluded from the study, the variability in the females would have been significantly reduced. The examination of a subset of the same mice repeatedly, over time was designed to help lessen the effect of this variability. Despite the variability, with measures of lesion volume, we were able to detect transient neuroprotection with hypothermia at p18 that is not present in the females at p30. In fact, median residual brain volumes in both normothermia and hypothermia treated females are the same at p18 and p30 as reflected in ≤0% growth in volume in both groups (Fig. 4) and consistent with the regional volume differences and poor performance in Y-maze testing by both female treatment groups. In future studies, body weight prior to HI should be collected, as this may affect temperature and response to injury [65]. It should be noted that core body temperature monitoring was not continued after pups returned to the nest in this study and may be affected by many factors such as nesting material, activity level, and stress [66]. Future studies should examine this data point as the effect of sex on post-HI/hypothermic temperatures is unknown and may differ. A further limitation of this work is that we did not include data from earlier time points after injury; however, to establish a relevant model, it was first necessary to demonstrate neuroprotection for an extended time following injury. Clearly, both earlier and later time points are now of interest in this model.

Development of a standardized mouse model will allow longitudinal evaluation of injury response to HI and hypothermia in neonatal mice. The longitudinal evolution of HI brain injury and response to hypothermia is meaningful to take advantage of: i) the inherent advantages of mice as an experimental tool to determine the genetic regulation of injury and treatment, ii) the ability to follow for extended periods of time after the acute phase of injury to quantify recovery and plasticity in response to injury and treatment, and iii) to tailor adjuvant therapies based on genetics and sex.

This study provides a modified neonatal mouse model of hypothermia following HI with examination of sex and region specific response to HI and hypothermia. Males in this model demonstrate sustained neuroprotection in behavior and imaging that females do not consistently demonstrate. Females demonstrate behavioral abnormalities after HI that are not improved with hypothermia and not explained fully by MRI measures of regional residual brain volumes. There may be a transient but important effect of injury and therapeutic hypothermia on the cerebellum. Continued examination of sex differences and outcomes in response to injury and treatment in both translational and clinical studies is essential.
