# Correction: Hypoxia-Ischemia and Therapeutic Hypothermia in the Neonatal Mouse Brain—A Longitudinal Study

**DOI:** 10.1371/journal.pone.0129406

**Published:** 2015-05-26

**Authors:** The PLOS ONE Staff

Table 1, “Percentage injury at p18 and p30”, incorrectly appears twice. Please see the corrected Table 1 here.

**Table 1:** Percentage injury at p18 and p30.

Sex	Region	Treatment Group	Median % Injury at p18 (Interquartile Range)	Significance (p)	Median % Injury at p30 (Interquartile Range)	Significance (p)
**Male**	Hippocampus	Control	7.10 (0.67–11.44)	p < 0.001*	1.09 (-0.89–6.17)	p < 0.001*
		Normothermia	73.33 (54.02–85.5)	—	67.07 (44.69–84.67)	—
		Hypothermia	14.6 (9.53–28.91)	p < 0.001*	29.99 (18.57–51.17)	p = 0.01*, p = 0.002†
	Cortex	Control	7.93 (3.68–11.01)	p = 0.001*	4.07 (2.38–6.49)	p = 0.008 *
		Normothermia	37.04 (24.67–53.59)	—	28.29 (15.34–43.72)	—
		Hypothermia	8.8 (4.09–14.9)	p = 0.001*	8.85 (2.21–23.6)	p = 0.05*
	Striatum	Control	3.56 (0.43–8.16)	p = 0.003*	2.02 (-1.19–7.35)	p = 0.04*
		Normothermia	34.84 (18.93–43.79)	—	20.62 (3.4–50.12)	—
		Hypothermia	14.46 (7.3–27.89)	p = 0.04*	18.11 (9.79–30.66)	—
	Thalamus	Control	0.00 (-0.21–0.47)	p = 0.002*	0 (-0.14–0.61)	p < 0.001*
		Normothermia	7.13 (4.83–12.33)	—	15.65 (5.19–20.68)	—
		Hypothermia	3.01 (-0.4–8.31)	p = 0.04*	7.25 (5.09–8.6)	p = 0.04*, p = 0.02†
**Female**	Hippocampus	Control	3.23 (0.3–5.05)	p = 0.04*	0 (-10.56–26.15)	p = 0.03*
		Normothermia	71.91 (15.8–96.92)	—	51.4 (33.72–70.55)	—
		Hypothermia	55.72 (23.35–81.08)	p = 0.06*	70.12 (48.9–81.82)	p = 0.005†
	Cortex	Control	8.31 (1.81–13.3)	p = 0.19‡	4.86 (0.76–6.8)	p = 0.07‡
		Normothermia	36.72 (9.8–53.19)	—	20.03 (10.4–35.49)	—
		Hypothermia	25.41 (3.06–47.6)	—	31.1 (18.53–50.65)	—
	Striatum	Control	0.26 (-3.05–7.66)	p = 0.04*	0 (-1.18–2.51)	p = 0.02*
		Normothermia	41.76 (20.59–56.19)	—	23.57 (14.29–40.18)	—
		Hypothermia	25.16 (7.29–43.57)	—	33.22 (16.67–51.02)	p = 0.01†
	Thalamus	Control	2.15 (-0.66–7.02)	p = 0.09‡	0.94 (-0.7–3.9)	—
		Normothermia	10.74 (5.28–22.4)	—	7.47 (3.64–10.9)	—
		Hypothermia	7.02 (4.63–10.75)	—	7.05 (4.02–16.26)	p = 0.02†

* = vs. NT

† = vs. ctrl

‡ = between groups
